# The Effect on Theatre Nurses for Rendering Perioperative Care to Patients Living with HIV in a South African Tertiary Hospital

**DOI:** 10.1155/2023/1889208

**Published:** 2023-09-17

**Authors:** Rudzani Ifodia Ngaledzani, Avhatakali Allga Ndou-Mammbona, Azwihangwisi Helen Mavhandu-Mudzusi

**Affiliations:** ^1^Department of Health Studies, University of South Africa, Pretoria, South Africa; ^2^Office of Graduate Studies and Research, College of Human Sciences, University of South Africa, Pretoria, South Africa

## Abstract

**Purpose:**

The study aimed to gain an in-depth understanding of how theatre nurses are being affected when they render perioperative care to patients living with HIV in a South African tertiary hospital.

**Background:**

There is a scarcity of studies that focus solely on the wellbeing of theatre nurses who render perioperative care to HIV patient due to the ramifications of the nurses' fear of contracting HIV. Patients living with HIV often receive substandard care.

**Objectives:**

To establish how theatre nurses are being impacted when rendering perioperative care to patient living with HIV, the study followed a qualitative approach using an interpretative phenomenological analysis design. Data were collected through in-depth individual interviews from ten theatre nurses who were purposively selected according to specific criteria. They voluntarily agreed to participate. An interpretive phenomenological analysis framework was used to analyse the data. Two main themes emerged from the data analysis, namely, the negative effect on nurses' wellbeing and the impact that it had on them professionally.

**Results:**

The study revealed that the perioperative care of patients living with HIV had a negative impact on physical, mental, and social wellbeing of theatre nurses. Their compromised wellbeing in turn led to poor patient care, which put nurses at risk of losing their jobs and even potentially having to face litigation. The study further indicated that nurses did not receive psychological support from the management which further affected their health and professional performance.

**Conclusion:**

The study proposes that theatre nurses rendering perioperative care to people living with HIV should receive proper training and support; staff shortages should also be addressed. There is also an urgent need for appropriate and sufficient protective equipment. Such changes will be essential in order to mitigate the negative impact that their jobs have on their wellbeing and on them in their professional capacity.

## 1. Introduction

This study focuses on theatre nurses and the effect on them for giving perioperative care to patients living with human immunodeficiency virus (HIV). The HIV pandemic is still a global concern which needs a concerted effort. This is evident in all the different programs geared towards combating HIV and AIDS. One of the latest programs targeting the ending of the AIDS pandemic is the UNAIDS 90-90-90 program with the (already past) target date of 2020 [1]. The aim of this program is for 90% of the global citizens living with HIV to know their HIV status; 90% of these should be on antiretroviral treatment (ARV); and 90% of these should have their viral load suppressed [2]. The progress towards achieving the UNAIDS targets differs from country to country. For example, South Africa is on its way to achieving the target of all people living with HIV knowing their HIV diagnosis but lagging in the remaining two 90s of the 90-90-90 targets [1]. One of the identified hindrances to the uptake of ARVs among HIV patients is stigmatisation by healthcare workers [3]. People living with HIV have named different types of behaviours reflecting this stigmatisation by healthcare workers. These behaviours include wearing extra protective clothing during medical procedures (such as extra gloves and double protective gowns during surgery) and refusing to perform some medical procedures on patients living with HIV. The same experiences were shared in a study conducted in one of the tertiary hospitals in South Africa [4] which found that nurses wore two or even three pairs of gloves as they were afraid of being infected by HIV while assisting with surgical procedures or caring for patients living with HIV.

There are several factors that contribute to the stigmatisation of and discrimination against people living with HIV among healthcare workers. These factors include individual, hospital, and systemic factors. The individual factors often include the healthcare worker's religious beliefs, age, and gender. Hospital policies and bureaucracies are the leading hospital factors in stigmatisation [5]. In this regard, Opoku et al. [6] have reported that some hospitals in Ghana turned away patients living with HIV with the excuse that they were not hospitals designated for HIV patients. According to a study conducted by Mahy et al. [7] across 13 countries, the percentage of people who were reportedly denied health services at least once in the preceding 12 months because of their HIV status ranged from 1.7% in Malawi to 21% in Peru and Tajikistan. Sometimes the lack of postexposure prophylaxis in hospitals makes healthcare workers reluctant to render care to patients living with HIV [8]. Even in hospitals where postexposure prophylaxis (PEP) is available, some nurses are still reluctant to care for patients living with HIV. Assumedly, this is due to inadequate knowledge on the use of PEP and how HIV is spread [4, 9]. Some healthcare workers' attitudes towards patients living with HIV are based on a limited understanding of the risk involved, and this may be related to their area of specialisation and cultural setting [10].

Healthcare professionals' continued stigmatisation of patients living with HIV has been a concern in global public health [11]. Healthcare providers' stigmatisation of and discrimination against people living with HIV contribute to the compromised quality of care rendered to this population [12].

The stigma of and discrimination against people living with HIV do not end only in general wards but also extend to special nursing units such as the theatre. Theatre nurses view their environment as stressful and not conducive to providing quality care to patients living with HIV [13]. Studies have indicated the reluctance of nurses to scrub for a patient living with HIV. This practice can be quite dangerous, especially if it is a patient for a caesarean section as the delay may lead to foetal or maternal death. Kaptain et al. [14] are of the view that studies conducted on the healthcare of patients living with HIV mainly focused on healthcare professionals and nurses in general wards, not on those in a specialised unit such as the theatre. This gap compelled the researchers to explore how the perioperative care that theatre nurses in a South African tertiary hospital given to patients living with HIV affects them.

## 2. Study Design

An interpretative phenomenological analysis (IPA), which is one of the qualitative designs, was used to gain an in-depth understanding of the effect on theatre nurses when they render perioperative care to patients living with HIV. The IPA design was considered more appropriate as it offered the researchers the opportunity to explore how theatre nurses themselves experienced the effect of providing perioperative care to patients living with HIV.

### 2.1. Setting

The study was carried out in one of the tertiary hospitals in the Tshwane district, South Africa. The hospital has twelve operating theatres with different areas of speciality.

### 2.2. Population

The population of this study was all the theatre nurses between 25 and 65 years of age, with two years and more experience of working in this hospital.

### 2.3. Sample and Sampling Size

The sample size for the study was ten professional theatre nurses which was determined by category saturation [8]. The criterion-based purposive sampling was used with the aim of recruiting participants with relevant experience and attributes related to the study purpose.

### 2.4. Ethical Consideration

The study was conducted guided by the principles of the Helsinki Declaration. The Department of Health Studies Research Ethics Committee (REC-012714-039 (NHERC) reviews the ethics proposal and granted the ethical clearance for the study (Ethics Clearance Number HSHDC/987/2020) on 5 June 2020. Permission was also obtained from the Gauteng Department of Health as well as the understudy to the Chief Executive Officer of the hospital. Informed consent was obtained from the participants who voluntarily participated in the study. To protect the hospital, its name was never mentioned. Pseudonyms were used throughout the study to ensure confidentiality and the anonymity of the participants.

### 2.5. Data Collection

Data were collected from 1 April to 30 June 2021. The researchers used an in-depth semistructured interview outlined in the interview guide. The interview guide was written in simple English that every theatre nurse would understand easily. A pilot study was conducted before the actual interviews by interviewing two other nurses who previously worked in theatre and did not form part of the participants. The researchers took field notes during the interviews and recorded the interview sessions with a cell phone recorder. Data saturation [8] was reached at participant number seven. However, the researchers continued to conduct interviews till participant number ten. The researchers transcribed all the recordings verbatim.

### 2.6. Data Analysis

The initial data analysis was conducted by the two researchers (first and second authors) individually guided by the steps of interpretative phenomenological analysis highlighted in Howard et al. [15]. Each researcher had fully engaged themselves with the data by listening to each recorded interview and reading the transcripts repeatedly. In the process of rereading, the researchers were noting down similarities in each transcript using the comment box alongside the document. The researchers went through all the notes in comment boxes using different colour highlighting to come up with various categories. Similar categories were merged into themes. Each researcher verified all the themes with an open mind to see if there were any new emergent themes. The researchers looked for patterns in the themes and divided them further into smaller themes. At this stage, the researchers renamed the themes. After this step, the researchers discussed their results. In places where they differed, they discussed it until they came up with one table of themes. The third author, who was also the study supervisor, reviewed the table of themes and provided some input and guidance regarding some of the themes. The process led to the final table composed of two superordinate themes, themes, and relevant subthemes.

### 2.7. Measures to Ensure Trustworthiness

Trustworthiness entails a set of criteria for evaluating the rigor of qualitative studies [16], as cited in [17] as follows: credibility, dependability, confirmability, transferability, and authenticity.

To ensure credibility the researchers used the interview guide and audio-recorded the interviews that were transcribed verbatim. The researchers kept records of the interview recordings; each of the recordings was transcribed verbatim and analysed by the two researchers independently while the third researcher acted as the quality assurer of the findings. To ensure confirmability, the researchers kept an audit trail from information from the sampling, the interviews, and the data analysis to the writing of the research report. The researchers ensured transferability by giving a sufficient description of the research study and methodology to ensure easy understanding and guidance for other researchers wanting to undertake a similar study. The researchers also ensured authenticity by interviewing theatre nurses with varied experiences, recording the interviews, and writing field notes.

## 3. Results

Results are presented in two sections: (i) demographic data and (ii) the impact of rendering perioperative care to patients living with HIV.

### 3.1. Demographic Data

Demographic data in qualitative research guide the reader in understanding the sources of the quotations.  Table 1 presents the demographic data of the theatre nurses who participated in the study. Pseudonyms are used instead of participants' real names to ensure confidentiality.

All the study participants were African females. Only one of the participants was below the age of 30. The majority (60%) of participants were above 40. All the participants had more than two years of experience working in theatre. Though they have been working in theatre for several years, 70% of them had a Diploma in Clinical Nursing Science, Health Assessment, Treatment, and Care, which are a specialisation in theatre nursing.

### 3.2. The Effect of Rendering Perioperative Care to Patients Living with HIV

This section describes the impact on theatre nurses for providing perioperative care to patients living with HIV. Two superordinate themes have emerged from data analysis: (i) negative impact on nurses' wellbeing and (ii) professional impact. Both superordinate themes have their own relevant themes and subthemes as presented in Table 2.

#### 3.2.1. Negative Impact on Nurses' Wellbeing

This superordinate theme describes the effect that the perioperative care of patients living with HIV has on theatre nurses. It was found that it affected their physical health, social wellbeing, and their mental health.

### 3.3. Physical Health

This theme focuses on the impact that scrubbing for HIV-positive patients has on the physical health of theatre nurses. It consists of the following subthemes: physical exhaustion and increased risk of contracting HIV.

#### 3.3.1. Physical Exhaustion

The study showed that participants were particularly exhausted during surgery due to the high incidence of complications that occur during surgery on HIV patients.

*“Patients with HIV complicates most of the time. You cannot be relieved as you are responsible for that patient, you must monitor everything given to the patient; if the patient needs to be reopened, you must assist again and you are exhausted and lose concentration.” (Rofhiwa)*

#### 3.3.2. Increased Risk of Contracting HIV

Besides physical exhaustion, participants were also prone to the risk of contracting HIV. Results indicated that limited supplies of personal protective equipment and a lack of concentration due to fatigue put participants at a higher risk. Participants showed that fatigue, lack of concentration, and lack of protective clothing made them more likely to make mistakes that predisposed them to needlestick injuries and put them at risk of contracting HIV.

*“We don't always have all sizes of gloves, most of the time you are compromised about gloves that are not your size, either they are too small or too big as it's difficult to handle sharps and instruments. Fluids like blood can slip inside big gloves and you end up with blood in your hands.” (Rinae)*

#### 3.3.3. Excessive Fear of Contracting Certain Conditions Based on What Was the Witness

The results indicated that participants were psychologically affected by what they observed during surgery. They associated whatever symptoms they have or feel with what they witnessed in theatre.

*“It is emotionally draining, scary, and very sad especially when you see some of the conditions which patients with HIV develop. I had a pap smear done while being allocated in gynaecological theatre because of these conditions that I witness every day, especially after observing that the patients coming to the theatre for gynaecological conditions and removal of the uterus because of cancer or uterine abnormalities are getting younger and younger; I am more scared. It has really affected me psychologically every time I bleed heavily during my menstruation period, I go to the gynaecologist just to check if everything is still fine.” (Vhulenda)*

### 3.4. Mental Health

This theme is about the impact on the mental health of theatre nurses rendering perioperative care to patients living with HIV. It is composed of the following subthemes: phobia of conditions witnessed, carnophobia (fear of eating red meat), stress, and emotional trauma.

#### 3.4.1. Phobia of Conditions Witnessed

Participants mentioned that working with HIV-positive patients who had serious physical problems which required surgery caused them to be always afraid, thinking that they would also develop the conditions they observed during surgery.

*“All theatre nurses need to be referred for counselling, especially after certain procedures, like the ones (where) they remove the abnormal growth which develops in HIV patients. For some of those conditions, though we are not informed about HIV patients, we conclude that the person is HIV positive as we have seen to the HIV-positive patients. Some procedures, even if it is a hysterectomy, but for people living with HIV, you find that the procedure becomes complicated and can affect them emotionally. Sometimes you imagine the things that happened to the patient happening to you.” (Vhothusa)*

#### 3.4.2. Carnophobia (Fear of Eating Red Meat)

Observing human tissue being removed in the form of organs really affected the theatre nurses psychologically. Participants indicated that they were psychologically affected by what they observed during surgery. When afterwards they saw red meat, they associated it with human tissue. It was also difficult to eat red meat as they somehow associated it with eating human tissue observed during surgeries.

“*There is this one patient who came for removal of genital warts. The warts were very big like cauliflower and were obstructing the vagina and the whole perineum. We ended up doing a vulvectomy, and since that day I don't eat beef.” (Shaleen)*

#### 3.4.3. Stress

Some of the procedures performed in theatre affected the participants so much that they ended up developing stress. Study results revealed that most of the participants were working under stressful conditions, and they carried that stress beyond their work. Because they lived in the same community as the patients, they were always reminded of the ordeal.

*“It is very traumatic. You take this patient like your own child. It is very painful. When you go home, you are heartbroken thinking like the team did not do enough. Just imagine an eighteen-year old losing the uterus because of profuse bleeding related to being HIV positive. On top of that, the child did not survive. It is not a nice experience.” (Mulwela)*

*“It really affects me because scrubbing for these HIV patients does not only end up in theatre, but we do also mix with them in the community where we live, or we meet in a mall, and it just reminds them that this is the nurse who removed my uterus, and I will not have any more children because of her.” (Muvhuya)*

#### 3.4.4. Emotional Trauma

Participants mentioned that they were emotionally very traumatised by their experiences while nursing these patients perioperatively. They indicated a lack of organisational support from the managers.

*“When we go to the managers to report our psychological and emotional trauma, they feel like you are not strong. But being in theatre, having to assist in evacuating a four-month foetus because the mother is HIV positive and can be allowed to terminate that baby at any time is emotionally traumatizing. At some stage, the manager reminded me that you said you wanted employment, but I did not know that I would assist in abortion procedures that are against my religion. The manager also told me that I must go back to theatre and continue working.” (Ndivhuwo)*

### 3.5. Social Wellbeing

This theme focuses on how offering perioperative care to people living with HIV affected the interaction of nurses with other people. The theme composed of three subthemes, namely, sympathising with patients living with HIV, discriminating against HIV-positive patients, and irritability potentially leading to fighting among the staff members.

#### 3.5.1. Sympathising with Patients Living with HIV

Results of the study indicate that patients living with HIV tended to complicate a lot, and the theatre nurses often sympathised with them. As a result, the participants became too attached to the patients and put themselves in the patients' shoes, which emotionally drained them.

*“There is a lot of psychological impacts. I will give you an example of a young patient who comes to the theatre for evacuation of the uterus and end up with the uterus being removed. It is so painful for the nurse. You feel that maybe as a team, we did not do enough to help this patient. It drains you emotionally because you invest your emotions and get attached to your patient as a nurse or a parent because most of these patients coming for this procedure are young. You put yourself in the boot of this patient when they wake up from anaesthesia, and it is frustrating.” (Rofhiwa)*

#### 3.5.2. Discriminating against HIV-Positive Patients

Participants mentioned that all patients who came to the theatre were being treated as potentially HIV positive. They protected themselves and always wore two pairs of gloves as they feared contagion.

*“All the patients that come to the theatre I treat them like they are suspects when they come into the reception area, I ask them if they are on chronic medication, and if they disclose that they are HIV-positive, I take extra precaution such as putting on two pairs of well-fitting gloves. I also become extra vigilant.” (Rinae)*

#### 3.5.3. Irritability Potentially Leading to Fighting among Staff Members

Participants mentioned that most patients living with HIV complicate intraoperatively. When things did not go as planned, the whole team started to panic and team members became very irritable with each other.

*“When the patient is bleeding, you must be fast. The more you are trying to be fast, the more chances of pricking yourself. You tend to be irritated by other team members, especially the doctors. If you are not as quick as they expect you to be, they get very irritated, and in turn, you are also irritated by the way they are shouting at you.” (Mulayo)*

## 4. Professional Impact

The study revealed that not only were theatre nurses' physical, mental, and social wellbeing negatively affected but they were also affected in their professional capacity. This is the second superordinate theme, and underneath it, we will discuss the two themes that emerged, namely, poor patient care and the threat of losing one's job, and the following subthemes flow from that: compromised healthcare services, an increased risk of making mistakes, a high possibility of litigation, and the risk of being struck from the professional register.

### 4.1. Poor Patient Care

The study revealed that patients living with HIV were not given holistic quality patient care. The principal problem was that theatre nurses were afraid of contracting HIV in the line of duty.

*“To be honest, everybody is afraid to scrub for an HIV patient. Chances of getting infected are very high, as the gloves sometimes will be torn while doing surgical procedure and if you have a cut you can end up being infected.” (Rofhiwa).*

#### 4.1.1. Compromised Healthcare Services

This subtheme highlights the quality of care associated with HIV-positive patients in a resource-constrained setting. One participant reported serious problems regarding supplies of consumables, including personal protective equipment and staff shortages.

*“The consumables are scarce, and it affects us emotionally as you will have to control all items and it causes burnout and I ask myself why the hospital is doing this to us, letting us work without protective equipment and clothes because these items must be readily available.” (Ndivhuwo)*

#### 4.1.2. Increased Risk of Making Mistakes

The study also found that working in theatre was exhausting due to the urgency of surgeries; it was an environment that was not conducive to quality care anyway due to a shortage of personnel as a result of high absenteeism and a lack or shortage of some personal protective equipment needed to care for people living with HIV. Due to these problems, theatre nurses often ended up making mistakes while nursing people living with HIV during surgeries. Study participants mentioned that because of exhaustion and compromise in wearing the wrong glove sizes, they were more at risk of making mistakes, including pricking themselves or leaving swabs or sharps in the abdomen.

*“Due to the urgency of procedure, medicolegal hazard can occur. One can miscount the swabs and leave some in the abdominal cavity. When the patient is bleeding, and one must be very fast and that is where most of mistakes happen. Another thing is that we are always tired because we are not resting enough.” (Vhushai)*

### 4.2. Threat of Losing One's Job

The results revealed that working in theatre was like risking one's future because one could be dismissed from work at any time as chances of making mistakes were very high. The theatre nurses worked under stressful conditions, and the patients they were dealing with were mostly HIV positive. Patients living with HIV needed constant monitoring which was difficult to do as there were many patients to look after whilst even sometimes being the only nurse. The following participant transcript attests to this:

*“It is not easy to work in theatre, it is always busy and most of the time we do not even go for lunch. The resources are always unavailable. I was working alone with the two doctors, and it was difficult to monitor patients after surgeries. The patients end up going to the ward without being properly monitored. I was also afraid that I may lose my job if anything went wrong. The other nurse I was supposed to work with was off sick.” (Mulwela)*

#### 4.2.1. High Possibility of Litigation

Apart from fear of being dismissed, there was also a high possibility of potential lawsuits from a patient or a relative of a patient who received inadequate care. Theatre nurses' fears were that if they were sued, they would end up forfeiting their pension as the state would take their money to pay the aggrieved litigant.

*“It is not easy, you can lose your pension in a split of a second, that is why it needs one to be always vigilant and alert when performing the surgical procedure. If anything, bad happens to a patient, as a nurse you are the first one to be blamed. There is no way they cannot find any mistake in the file of a patient, so it means you already lost the case before they can even report it to the South African Nursing Council.” (Rofhiwa)*

#### 4.2.2. Risk of Being Struck from the Professional Register

Theatre nurses indicated that due to compromised healthcare services and the risk of making mistakes, they had a high risk of being struck off the professional register. The study also revealed that there was too much responsibility and accountability when nursing patients living with HIV perioperatively. If they did not do their work with caution and professionally, there was a constant risk of being struck off the roll.

*“There is a procedure to be followed for relieving each other. But if a person relieves you in an emergency or in a patient that is not stable which is very common to HIV patients, really a lot of things can happen there, and you might find yourself in lawsuits when swabs are left inside the abdomen.” (Vhulenda)*

## 5. Discussion

Results indicate that working with HIV-positive patients during the perioperative stage has a negative impact on theatre nurses. In this regard, the study has identified two superordinate themes: nurses' wellbeing and their professional service.

### 5.1. Nurses' Wellbeing

Most of the participants were worried about the risk of contracting HIV while being at the workplace. They said they were being exposed to touching patients' body fluids because of limited personal protective equipment and impaired concentration due to exhaustion. Testimony to that is a systematic review conducted in America, which states that the lack of personal protective equipment predisposes nurses to transmittable infections, including HIV [18]. Some of the participants also reported being pricked by a needle during an operation on an HIV-positive patient and having had to take postexposure prophylaxis. This means that some of the participants might even have been infected with HIV, carrying the physical effects of living with HIV and its corresponding opportunistic infections. The risk of contracting HIV while working with patients living with HIV is a reality which has also been documented by Moshidi et al. [13], where participants mentioned that they accidently could have touched patients' body fluids, while delivering invasive or noninvasive medical intervention in an emergency. They thought that they would be safe from HIV exposure if they took precautions when in direct contact with the patient. An increased workload is one of the significant causes of physical exhaustion experienced by professional nurses. Ghasemi et al. [9] described how nurses were always exhausted by the way they lifted patients from one stretcher to the other perioperatively, as they did not want to be in touch with the patient's body fluid for the fear of contracting HIV.

Working with HIV-infected patients during the perioperative phase also had an impact on theatre nurses' emotional wellbeing. Participants mentioned that they were afraid. They feared developing the conditions they observed during surgery. When they saw red meat, for example, they associated it with human tissue. Asimah Ackah and Adzo Kwashie [19] also found that theatre nurses were psychologically affected by what they observed during surgery on patients living with HIV. Our study further revealed that most of the participants were working under stressful conditions, and they carried this stress beyond their work. As they lived in the same community with these patients, it always reminded them of the ordeal. Asimah Ackah and Adzo Kwashie [19] also found in their study, which explored the stress levels of theatre nurses in a Ghanaian teaching hospital, that most of the nurses were working under stressful conditions and that they carried this stress beyond their work. When things did not go as planned, the whole team started to panic and team members became very irritated with one another [19].

In our study, the theatre nurses indicated that they were emotionally traumatised by their experiences while nursing patients living with HIV perioperatively. They experienced a lack of organisational support from the managers. It was also found that participants became too attached to the patients and often put themselves in the patients' shoes, which drained them emotionally. They mentioned that all patients who went into the theatre were treated as potentially HIV positive, and they protected themselves and always wore two sets of gloves as they feared contagion. Sharing this same narrative is another study conducted in Ghana by Abdulai et al. [20] which found that nurses did not feel protected at all when nursing patients living with HIV. This was because the personal protective equipment they used was of poor quality. The gloves could tear at any time while one was busy with the surgical procedures. The results also indicated that the participants were psychologically affected by what they observed during surgery. Like in our study, the theatre nurses associated their own ailments and their conditions with what they witnessed in theatre. The same thing was found in the study conducted by Tantchou [21] that nurses were being affected psychologically by what they were witnessing in theatre while surgery was being performed on people living with HIV.

### 5.2. Professional Impact

Besides the issue of risk of contracting HIV and developing opportunistic infections, rendering perioperative care to HIV-positive patients had an impact on the theatre nurses' professional service. Participants indicated a serious problem with the compromised healthcare services being rendered to patients with HIV during the perioperative phase. Raymond et al. [22] are of the view that HIV-positive patients undergoing surgery are receiving substandard care in most public hospitals. The fact that patients usually bleed a lot during surgery is a big factor affecting the quality of care given to HIV-positive patients undergoing surgery as theatre nurses want to avoid contagion. Furthermore, the participants indicated that it was difficult to give quality care without adequate and sufficient protective material. There were similar findings in a study conducted by Mammbona and Mavhandu‐Mudzusi [10] where inadequate resources compromised the level of care provided to patients living with HIV.

Then, there is also the issue of professional and legal ramifications that may follow substandard care. Sometimes a minor operation such as a caesarean section may end up being a hysterectomy which the patient has not signed for. Some women even die during such surgery or postsurgery. Similar to the abovementioned findings is the study conducted in Burkina Faso [23] where women received a hemostasis hysterectomy without their knowledge. These are just examples of the possible general mistakes that can happen, let alone in caring for HIV-positive patients, where more hazardous factors make mistakes even more likely.

When theatre nurses have been involved in an overextended procedure due to complications, they become exhausted and lack of concentration often ensues. This can result in nurses making mistakes like leaving swabs or other instruments in the patient's abdomen. Ghobadian et al. [24] say that the most common error during abdominal surgery is leaving behind abdominal swabs and other surgical instruments inside the abdomen of a woman, which can lead to sepsis and then death, in some instances [25]. In all these incidences, nurses are expected to write an incident report which ends up being discussed in the hospital mortality meeting, or, in the case of South Africa, even sometimes being sent to the South African Nursing Council. Such incidents, if they realise that the nurse was somehow at fault, may lead to litigation and even to that nurse being struck off from the register as a professional nurse, which means the end of their professional life. The same thing was found in the study conducted by Wielogórska and Ekwobi [26], where nurses were affected professionally by being struck off the roll if found to have been at fault while assisting doctors during surgeries. The participants in this study indicated that the risk of medicolegal hazards became even higher when providing care to HIV patients in a resource-challenged hospital.

Another important issue mentioned by the nurses in the study was that one would also possibly forfeit one's pension if one was dismissed and found to have been at fault, with one's pension money being used to reimburse the victim. This was clearly a big potential threat to the participants, something that might have affected them psychologically as well. All in all, our study showed that there was too much responsibility and accountability placed on theatre nurses when rendering perioperative care to HIV-positive patients with their problems and concerns not being adequately addressed.

### 5.3. Limitation

The researcher used convenience purposive sampling, and participants came from only one hospital, so the sample may not be representative of the healthcare population in other hospitals and other categories of professional nurses. This is unfortunately unavoidable in qualitative research as the focus is of contextual relevancy, and the IPA design focuses on case per case rather than generalising the findings. However, the researchers provided relevant information about the study, including the participants, sampling, data collection, and data analysis, which can assist with the transferability of the findings to other similar settings. The study was conducted during the COVID-19 lockdown, and because of having had to wear masks and social distancing, it was difficult to observe some of the nonverbal cues as the participants' mouths and noses were covered. It was also difficult to maintain eye contact with all the participants as some of them put on face shields. This might have affected the probing process.

## 6. Conclusion

There is evidence that offering perioperative care to patients living with HIV has a negative effect on the physical, mental, and social wellbeing of theatre nurses, while it also impacts them in their professional capacity. They may end up being caught up in a vicious cycle of becoming exhausted (due to diminished wellbeing) and then accidently making some mistakes when rendering care, which in turn may lead to litigation. Furthermore, a nurse who may have a constant underlying fear of losing her job and possible litigation may become very anxious which may in turn predispose them to making mistakes and concomitant physical challenges, including the possibility of being infected with HIV.

To prevent all this negative impact, the researchers recommend proper in-service training of nurses especially in relation to people living with HIV. The government/hospital should increase the staff working in theatre to ensure that nurses have adequate time to relieve one another and get adequate rest. There should be debriefing and counselling of theatre nurses especially after complicated procedures which might have led to loss of life or irreversible damage to the patient. Though there is speciality allowance for the nurses who have advanced training in theatre technique, there is a need for offering a danger allowance to all nurses who are working in theatre because they would inevitably be rendering care to HIV-positive patients. There is also a need for providing appropriate and sufficient protective equipment. The implementation of such recommendations may assist in lessening the negative effect that rendering perioperative care to patients living with HIV has on theatre nurses. In turn, very importantly, this will contribute to the improvement of healthcare for people living with HIV [27].

## Figures and Tables

**Table 1:** Demographic characteristics of participants.

Participants	Age range	Experiences in years	Theatre nursing specialisation
MULAYO	30–39	03	No
MUVHUYA	30–39	13	Yes
MULWELA	40–49	20	Yes
SHALEEN	20–29	03	No
VHOTHUSA	50–59	09	Yes
VHUSHAI	40–49	08	No
VHULENDA	50–59	10	Yes
RINAE	50–59	20	Yes
ROFHIWA	50–59	20	Yes
NDIVHUWO	30–39	06	Yes

**Table 2:** Themes.

Superordinate theme	Themes	Subthemes
Negative impact on nurses' wellbeing	Physical health	Physical exhaustion
		Increased risk of contracting HIV
		Psychosomatic disorders
	Mental health	Phobia of conditions witnessed
		Carnophobia (fear of eating red meat)
		Stress
		Emotional trauma
	Social wellbeing	Sympathising with patients living with HIV
		Discriminating against HIV-positive patients
		Irritability leading to potential fighting among staff members

Professional impact	Poor patient care	Compromised healthcare services
		Increased risk of making mistakes
	Threat of losing one's job	High possibility of litigation
		Risk of being struck from professional register

## Data Availability

The datasets used and/or analysed during the current study are available from the corresponding author on reasonable request.
